# Recent Advances of Studies on Cell-Penetrating Peptides Based on Molecular Dynamics Simulations

**DOI:** 10.3390/cells11244016

**Published:** 2022-12-12

**Authors:** Jun Ouyang, Yuebiao Sheng, Wei Wang

**Affiliations:** 1 School of Public Courses, Bengbu Medical College, Bengbu 233030, China; 2 Collaborative Innovation Center of Advanced Microstructures, National Laboratory of Solid State Microstructure, Department of Physics, Nanjing University, Nanjing 210093, China; 3 High Performance Computing Center, Nanjing University, Nanjing 210093, China

**Keywords:** cell-penetrating peptides, molecular dynamics simulations, peptide-lipid interaction, internalization mechanism, CPP decoration and coupling

## Abstract

With the ability to transport cargo molecules across cell membranes with low toxicity, cell-penetrating peptides (CPPs) have become promising candidates for next generation peptide-based drug delivery vectors. Over the past three decades since the first CPP was discovered, a great deal of work has been done on the cellular uptake mechanisms and the applications for the delivery of therapeutic molecules, and significant advances have been made. But so far, we still do not have a precise and unified understanding of the structure–activity relationship of the CPPs. Molecular dynamics (MD) simulations provide a method to reveal peptide–membrane interactions at the atomistic level and have become an effective complement to experiments. In this paper, we review the progress of the MD simulations on CPP–membrane interactions, including the computational methods and technical improvements in the MD simulations, the research achievements in the CPP internalization mechanism, CPP decoration and coupling, and the peptide-induced membrane reactions during the penetration process, as well as the comparison of simulated and experimental results.

## 1. Introduction

Cell-penetrating peptides (CPPs), also named protein-transduction domains (PTDs), are usually amphipathic and/or cationic molecules consisting of 5~30 amino acid residues, and rich in basic amino acids such as arginine and lysine. They exhibit their biological activity by interacting with cellular membranes, and belong to the category of membrane active peptides (MAPs) as well as antimicrobial peptides (AMPs). Such peptides are typically unstructured in aqueous solutions, but fold into a well-defined secondary structure near the membrane [1]. Compared to AMPs, CPPs can penetrate cellular membranes without disrupting their integrity. Furthermore, CPPs can not only cross cell membranes, but also transport covalently or non-covalently linked bioactive substances, such as proteins [2,3], peptides, DNA, RNA, fluorescent labels, nanoparticles [4], viruses, liposomes [5,6], oligonucleotides [7], and drugs, into the cells. Regarding the capacity of delivering bioactive active substances into the eukaryotic cells, CPPs exhibit brilliant prospects in the field of therapeutics delivery [8]. Although CPPs can promote the delivery of cargo molecules, the nonspecific characteristics of CPPs themselves to cells may lead to a decrease in the specificity of the CPP-cargo complex and an increase in cytotoxicity. How to target CPPs and cargo molecules to specific cells effectively while maintaining their biological stability and biocompatibility in complex environments has always been an active area of research in the pharmaceutical realm. In recent years, a great number of studies have reported the potential of CPPs as carriers for the treatment of various diseases, such as cancers [9,10,11,12,13,14], diabetes [15], and Alzheimer’s disease [16]. It has been found that some CPPs have exhibited greater selectivity for cancer cells in preference to noncancer cells [17]. For example, poly-L-arginine could facilitate the cellular uptake of doxorubicin and increase the cytotoxic effect of doxorubicin in human prostate cancer DU145 cells [18], and polyarginine (R11) conjugated with fluorescein isothiocyanate FITC-R11 has also been proved to be a more specific molecular probe for bladder cancer and has shown potential applications as imaging agents in cancer treatment [19].

Over the past 30 years, the outstanding properties of CPPs have attracted considerable attention and significant progress has been achieved. A variety of experimental methods, such as nuclear magnetic resonance (NMR) experiments, circular dichroism (CD) spectroscopy, isothermal titration calorimetry (ITC), single-molecule fluorescence microscopy, fluorescence spectroscopy, dynamic light scattering, and lamellar neutron diffraction, have been used to explore the internalization process, penetration mechanism and potential applications of CPPs. Even so, differences in experimental strategies, experimental conditions, and analytical methods may result in contradictory results. In addition, current experimental techniques cannot provide atomic information with enough temporal and spatial resolution about the internalization process of CPPs. As a consequence, the uptake mechanism of CPPs is still unclear despite having a host of alternative models.

**Figure 1:**
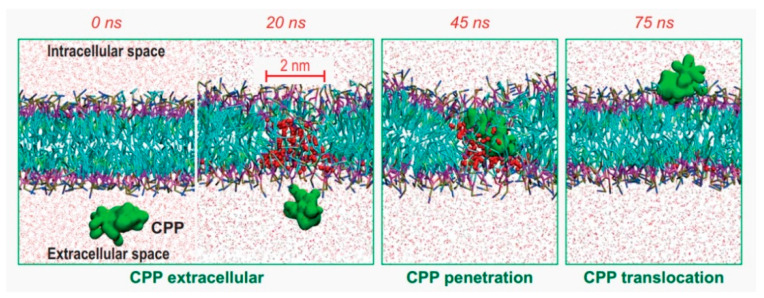
Coarse-grained simulations show that the hyperpolarization favors the formation of ~2-nm-wide water pores, which would be used by CPPs to directly translocate across membranes within a few tens of nanoseconds. Membrane hyperpolarization was achieved by setting an ion imbalance through a net charge difference of 30 positive ions (corresponding to a $V_{m}$ of ~2 V) between the intracellular and extracellular spaces. The CPPs used in the simulations were TAT-RasGAP_317–326_, TAT, R9 and Penetratin, respectively, and the simulation system also contained a natural cell membrane-like composition (for both inner and outer leaflets). Image reprinted from Ref. [25].

With the development of computational resources and methods, molecular dynamics (MD) simulation has become a powerful tool to explore the internalization mechanisms, which can provide kinetic clues to peptide–membrane interactions and cellular uptake processes at the atomistic level [20,21,22,23,24]. In addition, we can monitor various effects during the process of penetration by changing simulation conditions such as temperature, pH condition, initial conformation or orientation of the peptides, and the lipid composition of the membrane. For example, by decreasing the transmembrane potential, Trofimenko et al. coined a megapolarization situation, which could trigger the formation of water pores, and enable the direct translocation of CPP into cells [25] (Figure 1). As an effective complement to the experimental results, MD simulations have been used to study the CPPs with great success.

In this review, we begin with a brief introduction to the general properties of CPPs, including the CPP families, categories and cellular uptake mechanisms. Then, we describe the progress of MD simulations on CPPs from four aspects, including the computational methods and technical improvements in MD simulations, the research achievements in the uptake mechanism, the decoration and coupling of CPPs, and the influences and reactions of membrane models during the penetration process.

## 2. Categories and Cellular Uptake Mechanisms of CPPs

Since the discovery of the first CPP in 1988, thousands of CPPs have been discovered or designed artificially. CPPsite 2.0 [2], the database of cell-penetrating peptides, contains a total of 1855 entries of CPPs along with their secondary and tertiary structures now. Some of these CPPs have been well studied. Taking the HIV-1 Tat peptide (GRKKRRQRRRPPQ) as an example [26,27], the peptide contains eight positively charged residues and has no regular structure both in solution and on the membrane. However, it has been found that this kind of peptide can facilitate the uptake of large, biologically active molecules into the mammalian cells, and can mediate the delivery of different cargoes into tissues throughout living organisms. Nowadays, the Tat peptides have been successfully used for intracellular delivery of a broad variety of cargoes, including various nanoparticulate pharmaceutical carriers (liposomes, micelles, nanoparticles) [28,29]. Another typical example is penetratin (RQIKIWFQNRRMKWKK) [30,31,32], which is derived from the third helix of the Drosophila Antennapedia homeodomain. Penetratin has seven positively charged residues, and can penetrate directly into the giant monolithic vesicles [33]. It is worthy to note that both Tat and penetratin are arginine-rich peptides, suggesting that the arginine plays an important role in the uptake mechanism due to the bidentate hydrogen-bonding interaction between the guanidinium groups of arginine residues and the phosphate groups in the membrane, e.g., the polyarginine sequence (Arg8) has been demonstrated to be sufficient to drive molecules into cells [34]. Besides arginine, lysine also plays an important role in the CPP penetration process, such as Transportan 10 (TP10) (AGYLLGKINLKALAALAKKIL) [35,36], which is a lysine-rich chimeric CPP and adopts an α-helical secondary structure on the surface of membranes. Compared to Tat and penetratin, TP10 has more helical structure and shows higher efficiency in cellular uptake as the increase in CPPs’ helicity can promote the ability of cell-penetration. Since the previous findings, more and more CPPs have been designed to facilitate the penetration of cells across membranes into the cytoplasm.

**Figure 2:**
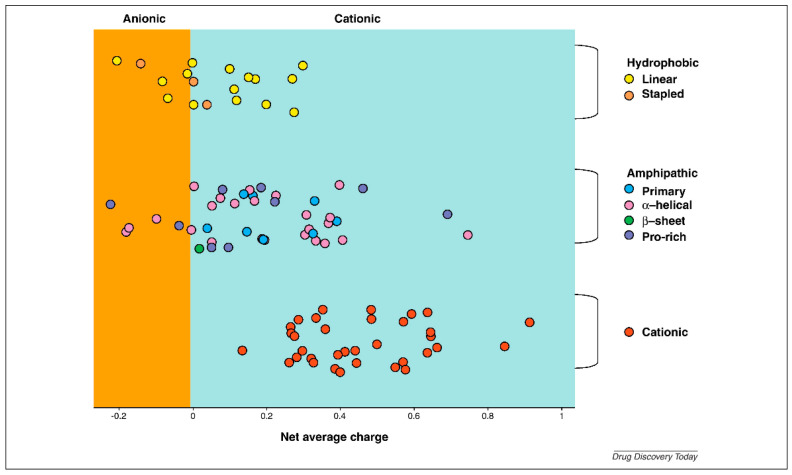
Distribution of CPPs by net average charge and category. Most of the CPPs have net positive charges. Many cationic CPPs are highly charged peptides, without any amphipathic arrangement or hydrophobic character. Anionic CPPs do not form a separate class but can be classified as hydrophobic or amphipathic CPPs. Image reprinted from Ref. [45].

As we know, CPPs have a common ability to penetrate cell membranes, although they are different in length, electric charge, hydrophobicity/hydrophilicity, flexibility, and so on. There are a variety of methods for classification of the CPPs [37]. The first one was based on the origin of the CPPs. In this manner, CPPs are classified into natural CPPs, designed CPPs and CPPs derived from peptide libraries. The natural CPPs are derived from natural proteins or peptides, such as the HIV-1 Tat peptide which is derived from the transcription transactivating protein of HIV-1 and the penetratin derived from the Antennapedia homeotic transcription factor. The designed CPPs are designed according to specific requirements or to mimic the structures of the known CPPs. A typical example for designed CPPs is transportan, which was designed by fusing amphipathic peptide mastoparan from bee venom to the fragment of human neuropeptide galanin. In recent years, a number of design methods were proposed to further enhance CPPs’ properties through integrating several different protein fragments into chimeric sequences, or incorporating of unnatural amino acids into peptides, and create numerous efficient CPPs, such as CADY (GLWRALWRLLRSLWRLLWRA) [38] and MAP (KLALKLALKALKAALKLA) [39]. Due to the lack of quantitative understanding of the sequence-structure-function relationship of CPPs and their complex mechanisms of action, it is difficult to achieve rational design and optimization of them. One possible approach is to obtain CPPs from the peptide libraries (large, diverse collections of related molecules) through high-throughput screening for desirable properties, such as targeting and delivery. The second classification method is based on the physicochemical properties (Figure 2), and CPPs can be divided into the cationic, amphipathic and hydrophobic ones. The cationic CPPs, such as HIV-1 Tat, penetratin and the Arg9 (RRRRRRRRR) [40] peptide, contain usually both arginine and lysine residues, and carry net positive charges which are important for the first contact with the membrane. The amphipathic CPPs that have both hydrophilic and hydrophobic regions, such as transportan, MAP and pVEC (LLIILRRRIRKQAHAHSK) [41], are the most numerous category, accounting for more than 40% of the known CPPs to date. They are strongly associated with neutral and anionic membranes, and their transmembrane transport patterns are closely related to their interactions with polar head and lipid chain regions of the membranes. The amphipathic CPPs can be further divided into two subclasses: primary amphipathic CPPs and secondary amphipathic ones [42]. The primary amphipathic CPPs normally contain more than 20 amino acids, have sequentially hydrophobic and hydrophilic domains along their amino acid sequence, and bind to both neutral and anionic lipid membranes with strong affinity, such as Pep-1 (KETWWETWWTEWSQPKKKRKV) [43] and TP10. They can penetrate into membranes with both high and low anionic lipid ratios and induce membrane leakage at low micromolar concentrations. The secondary amphipathic CPPs, such as MAP and penetratin, show a stronger affinity to anionic membranes than electrically neutral membranes. They show their amphipathic property through the α-helical or β-sheet folding, i.e., the distribution of their hydrophobic and hydrophilic domains occurs through the secondary structure formation. For example, penetratin has a random coil conformation in aqueous solution, while upon interacting with the anionic lipids it can form the α-helical or the β-sheet amphipathic secondary structures at lower or higher peptide-to-lipid ratios, respectively. As for the hydrophobic CPPs, such as K-FGF (AAVLLPVLLAAP) [44], they contain only apolar residues, or few charged amino acid residues in the sequence. The internalization mechanism of the hydrophobic CPPs are less well studied, but there is an opinion that their hydrophobic sequences are often critical for CPPs to penetrate cellular membranes. The third classification method is based on the CPPs’ cytotoxicity, which divides CPPs into the non-toxic and the antimicrobial ones. As with other chemical substances, the cytotoxicity of the CPPs may depend on the concentration, the amino-acid sequence, the composition of the membrane and so on. In addition, CPPs can also be classified as the nonspecific or the targeted ones depending on the capacity of cellular uptake. How to effectively target CPPs and cargo molecules to specific cells has always been a topic of interest in the area of drug development.

**Figure 3:**
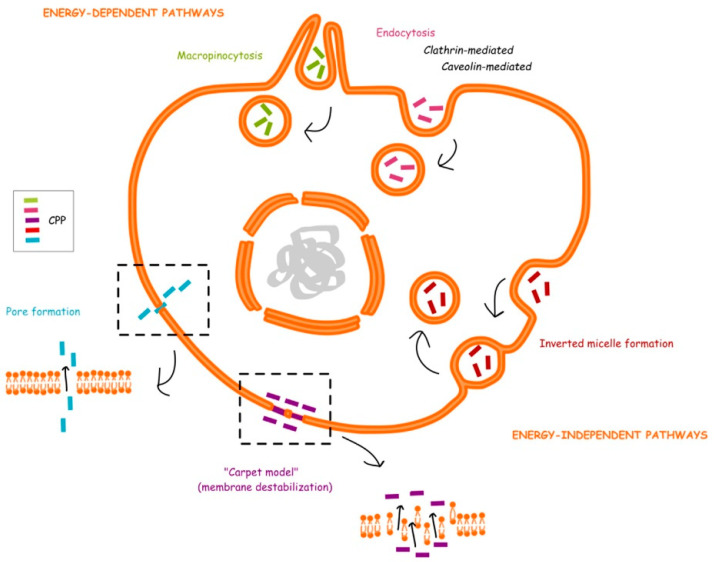
Mechanisms of CPP uptake across the cellular membranes. The cellular uptake mechanisms are mainly divided into two categories: energy-dependent endocytosis and energy-independent direct translocation. Image reprinted from Ref. [12].

Although CPPs can penetrate biological membranes in a variety of ways, the uptake and internalization mechanisms have not been well understood [46]. In general, the cellular uptake mechanisms can be divided into two categories: energy-dependent endocytosis and energy-independent direct translocation (Figure 3). For the first category, there are three main pathways of endocytosis, namely macropinocytosis, caveolae-mediated endocytosis, and clathrin-mediated endocytosis [47]. Interestingly, some studies have found that many CPPs cross cellular membranes by endocytosis at low concentrations, while at high concentrations they penetrate membranes through the direct translocation mechanism [48]. For example, as the peptide concentration increases, the way pTat and R8 peptides enter HeLa cells switches from endocytosis to direct translocation [49]. Although the hydrophobic core of biological membranes is the major barrier to charged CPPs, when the peptides aggregate on the membrane surface, the interactions between the positive charged CPPs and negative charged membrane components result in the lipid flipping or in pore formation, and CPPs subsequently penetrate the hydrophobic lipid core of the membrane with limited toxicity, suggesting that direct penetration is an ideal approach for designing intracellular delivery vectors. The transition of uptake mechanisms from endocytosis to direct penetration can also be evoked by lowering the temperature. For example, it did not abrogate HIV-1 Tat uptake by knock-down of clathrin-mediated and caveolin-mediated endocytosis, or even by incubation of the cells at 4 ℃ [50], suggesting that the Tat peptides penetrate the membrane directly in this case, and the direct translocation mechanism of CPPs is related to the instability of membrane to some extent. There are a host of models to explain the direct translocation, such as the pore formation model [51,52], inverted micelle model [53,54], carpet model [55,56], and membrane thinning model [57]. It is worth mentioning that there is no unified conclusion about the uptake mechanisms of CPPs, and it is agreed that the cellular uptake pathway depends on many factors, including the physicochemical properties such as charge distribution and peptide length [58], the secondary structures of peptides [1], CPP concentration [59], cell type [60], lipid composition of the membrane [61], the ability of CPP to interact with the membrane, the property of the cargo molecule and its coupling type with CPP [62], experimental conditions [63], environmental factors like pH condition and ambient temperature [59].

## 3. Techniques for CPP Simulations

The earliest MD simulations were used in 1957 to study the motion of a simplified hard-sphere system [64]. In 1964, Rahman simulated the systems of 864 Ar atoms with the Lennard-Jones potential [65], obtained the radial distribution function, diffusion constant, velocity autocorrelation function, and mean azimuth shift of the system, and compared them with experimental results. Over the decades, molecular simulation methods have made great progress with the development of simulation algorithms, force fields and computational resources. MD simulations allow us to rigorously control the composition and condition of the system, monitor the evolution of the system, and analyze the interactions at the atomic or molecular level. Thus, through the detailed simulations, we can obtain lots of microscopic details for CPP internalization by monitoring various indicators [66] such as evolution of the peptide conformation and orientation, time dependence of the distance and the contact area between peptide and membrane, depth of insertion of the peptide into the membrane, radial distribution functions, density profile of the system, charge distribution, deformation and reorganization of the lipid bilayer induced by the peptide insertion. Moreover, we can also obtain accurate peptide–membrane interactions and free energy landscape by analyzing hydrophobic/hydrophilic properties, electrostatic interactions, hydrogen bonds, interaction energies, the potential of mean force (PMF), etc. MD simulations can complement our understanding with information that is hard to gain from experiments, and excite new ideas on experiments to further examine the findings from simulations. Nevertheless, we should also keep in mind that the results of MD simulations depend to some extent on initial conditions, degree of equilibrium, system size, and the conditions under which the simulations are performed.

### 3.1. All-Atom MD Simulations, Coarse-Grained Simulations and Implicit Membrane Models

**Figure 4:**
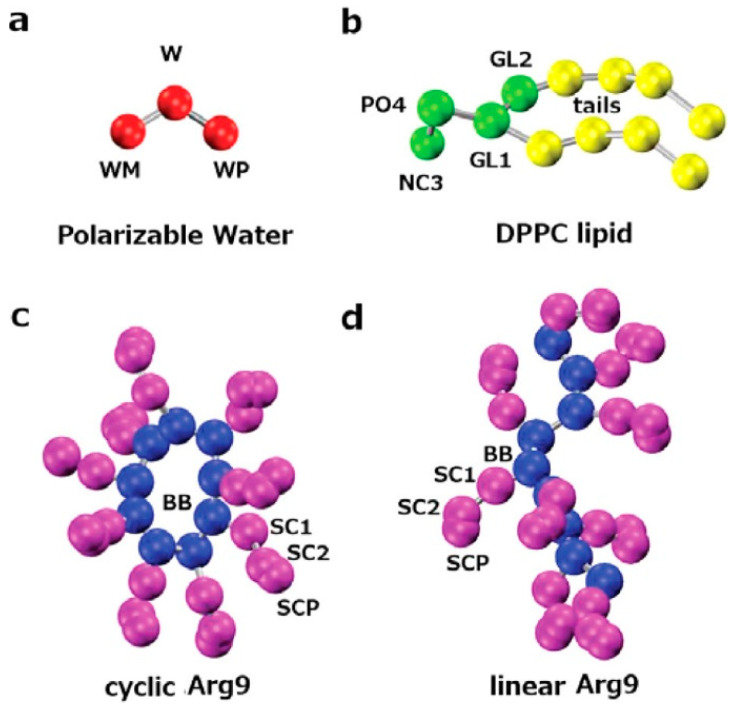
Bead representation of coarse-grained (**a**) polarizable water; (**b**) DPPC lipid; (**c**) cyclic Arg9; and (**d**) linear Arg9 molecules. All of the water beads are red. W, WP, and WM are neutral, positively, and negatively charged beads of polarizable coarse-grained water. The tails and head groups of coarse-grained lipid molecules are yellow and green. The NC3, PO4, GL1, and GL2 on lipid molecule are marked for choline, phosphate, and two carbonyl beads. The backbone and non-backbone beads of coarse-grained Arg9 peptide are blue and purple. The marked strings such as BB, SC1, SC2, and SCP correspond to the backbone, two non-charged beads, and the positively charged beads, respectively. Image reprinted from Ref. [67].

In general, MD is a method to simulate molecular motion based on Newtonian mechanics. The forces acting on each atom can be obtained according to the molecular potential function, and by given the initial velocity and position, we can obtain the trajectory of particles as a function of time. MD simulation can provide dynamic and thermodynamic information of the system in atomistic detail, and has become an effective tool for understanding the structure and function of biosystems. Classical all-atom MD simulations have higher resolution but are very time-consuming and computational cost expensive. In order to decrease the computational costs, improve the sampling efficiency and speed up the computation, some researchers adopt the coarse-grained models to perform the simulations. For example, Hu et al. explored the dependence of translocation free energetics on cyclic and linear Arg9 peptide conformation using coarse-grained MD simulations with the MARTINI force field (Figure 4) [67]. The results were consistent with the all-atom force field simulations, showing that the current coarse-grained force field provides sufficiently robust and reliable free energy differences. For coarse-grained simulations [68], groups of atoms are mapped onto different interaction beads, this allows the simulations to scale up to larger systems and longer timescale, but at the expense of the model accuracy, some behaviors and properties, such as the conformational transition of peptides, the protein folding events, the directionality of hydrogen-bonding patterns that underlies protein conformational stability, will be difficult to be investigated [69]. Given the balance between computational cost and simulation accuracy, another option is to consider implicit membrane models. Implicit membrane models attempt to account for the influence of lipids and water through the solvation free energy term in the energy function, which have many advantages such as fast speed, rapid equilibrium, and extensive exploration of configurational space. One of the problems with the implicit membrane models is that these models are only suitable for plane membranes because the membrane cannot be deformed, which introduces flaws to peptide–membrane interaction studies. To overcome this shortcoming, Lazaridis et al. incorporated the data from both all-atom simulations and experiments, and estimated the elasticity of lipid membrane by improving the implicit membrane model IMM1 and adding the free energy of membrane deformation [70]. For a helical secondary structure, the minimum energy pathways and transition states were determined by mapping the effective energy of the peptide as a function of vertical position and tilt angle. They used this model to study the transport of peptides, such as S4 helix, TP10W and DL1, across the membrane bilayers, and obtained results consistent with the experiment. In addition, the free energy predicted by IMM1 for the transfer of amino acid sidechains from bulk water to the membrane center was quite close to the calculated results based on all-atom simulations. However, the shape of the free energy profile of the charged side chains needed to be modified to reflect the all-atom simulation results. Later, the same group extended the implicit membrane model 1 (IMM1) to spherical and tubular membranes [71]. The expanded IMM1 was tested in a series of systems, including the wild-types and mutants of the antimicrobial peptide magainin, and the results were qualitatively consistent with experimental results.

### 3.2. Enhanced Sampling Approaches

**Figure 5:**
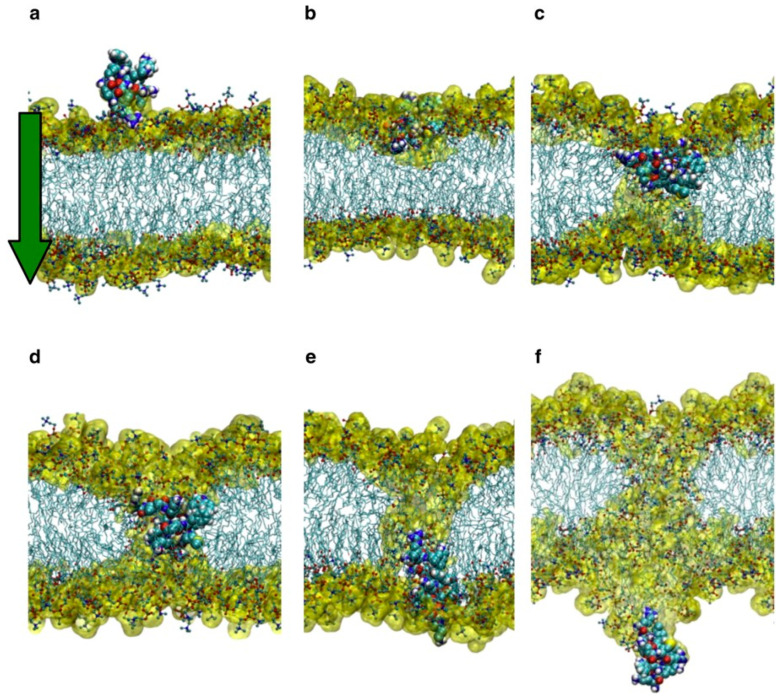
Key stages during the pulling of a single penetratin molecule through a DPPC bilayer: (**a**) the initial system; (**b**) the peptide binds to the upper monolayer; (**c**) the water-filled pore is formed; (**d**) the peptide reaches the center of the bilayer; (**e**) the peptide reaches the surface of the lower monolayer; and (**f**) the peptide is pulled out of the membrane. The arrow indicates the direction of the pulling force. The semitransparent yellow surface corresponds to the lipid-water interface. Image reprinted from Ref. [73].

Despite MD simulations providing an effective method as a special complement to the experimental approach, there are still many limitations, such as the difficulty of adequate sampling of energy landscapes, over-simplification of the membrane model, and limited accuracy of the force fields. Not only that, but the time it takes for CPPs to spontaneously translocate through the cell membrane is on the order of minutes, which is almost impossible to achieve in MD simulations unless the internalization process is accelerated artificially. Therefore, in the studies of the peptide–membrane system, some enhanced sampling approaches are often employed, such as steered MD (SMD), umbrella sampling, metadynamics, and replica exchange methods. SMD usually accelerates the process by providing an external steering potential or force to move the system along a specified path [72], and in peptide–membrane system, SMD is commonly used to pull the peptides across the membrane to speed up the penetration process. For example, Yesylevskyy et al. used a harmonic potential with a force constant of 1000 kJ/mol/nm^2^ to pull a single penetratin molecule through a DPPC bilayer to mimic the translocation of penetratin [73] (Figure 5). The umbrella sampling method can calculate the PMF along the reaction coordinate by the weighted histogram analysis method (WHAM) based on the data from a series of windows (each of which performs an MD simulation) [74,75]. Metadynamics can reconstruct the free energy surface as a function of few collective variables (CV) by driving the system to keep away from previously visited areas and to sample the unfavorable regions of the free energy landscape by a history-dependent bias potential along the CVs [76,77]. Replica exchange molecular dynamics (REMD) is a method which combines MD simulations with a Monte Carlo algorithm [78]. In REMD simulations, multiple replicas of the same system are simulated in parallel, with occasional exchange of temperatures or interaction energies between adjacent copies. In this way, REMD can overcome high-energy barriers and sample the conformational space sufficiently [79,80,81]. Another method to calculate the free energy surface of the reaction coordinate system using non-equilibrium dynamics is adaptively biased molecular dynamics (ABMD) method [82], which is characterized by the need for fewer control parameters, the flooding timescale and the kernel width, as well as the favorable O(t) scaling of molecular dynamics time. Therefore, it is suitable for long-term biomolecular MD simulation. The ABMD method also allows extensions based on replica exchange, which greatly improves the speed and accuracy of the method. Gimenez-Dejoz et al. used ABMD to determine the free-energy landscapes for the CPPs containing unnatural amino acids crossing membranes [83]. These simulation techniques mentioned above can provide information on the energetics and dynamics of various stages of CPP penetration, such as peptide binding to the water-lipid interface, peptide insertion into the membrane, and pore formation on the membrane. Another noteworthy approach is the Markov state models (MSMs) [84]. They are commonly used to determine events such as membrane penetration or translocation from long unbiased simulations, especially when good order parameters are not known a priori, and are therefore suitable for molecular models of CPP-membrane interactions. The MSM is mathematically expressed as an n×n transition probability matrix, where the entry in row i and column j represents the probability of transitioning from state i to state j within the lag time. If the system is in one of n discrete substates, after a fixed time, the system will be found in any of these n discrete substates. Thus, the long-term statistical dynamics of molecules can be modeled as a Markov chain on a discrete state space, and by running many simulations of short trajectories from each state, the transition rates between states can be precisely determined, resulting in long-term dynamical information [85,86]. MSM has been widely used to study a variety of biophysical problems, such as protein dynamics [87], protein folding [88], protein–ligand association [89], molecular self-assembly and aggregation [90]. For example, Xu et al. demonstrated that MSM analyses could be used to delineate the variation of free energy during the self-assembly process of a typical amphiphilic DPPC lipid [91].

Since umbrella sampling restraints the peptide through successively pulling, the calculated free energy should be considered as free energy along a particular path. Although it is difficult to obtain a full free-energy profile, comparisons between free energies along different paths can still shed insight on the translocation mechanism. Huang et al. calculated the free energies of translocating Arg9 into a neutral DOPC lipid bilayer along two different paths using the umbrella sampling MD simulations [92], and the results showed that the free-energy barrier along the pore path is 80 kJ/mol lower than the one along a pore-free path.

For the enhanced sampling techniques, the key factor in effectively obtaining the free energy profile is to choose a good CV. Kabelka et al. developed a new CV that can describe peptide insertion, local membrane deformation, and the internal degrees of freedom of the peptide associated with its charged groups [93]. By comparing different CVs, they demonstrated that all of these components are necessary to accurately describe peptide translocation, and the developed CV can reliably and effectively calculate the free energy of peptide translocation.

## 4. Simulations on CPP Internalization Mechanisms

**Figure 6:**
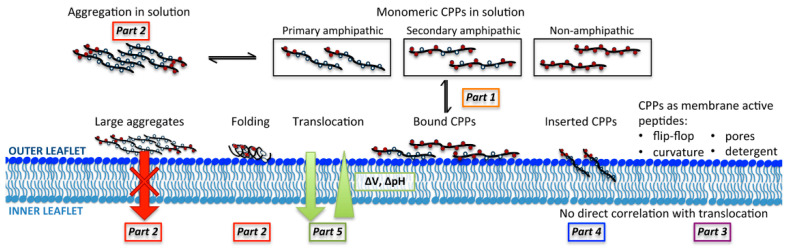
Different mechanisms of CPPs interacting with the membrane. Image reprinted from Ref. [46].

In order to better develop and optimize CPP application strategies, it is important to understand the cellular uptake mechanisms of CPPs. Although all CPPs share some common properties of cell penetration, different CPPs have different interaction partners with membranes, different membrane responses induced by peptide insertion, and different translocation mechanisms [94] (Figure 6). The interactions between CPPs and the membrane may come from various contributions such as electrostatic interactions, hydrophobic effects, and hydrogen bonding. In addition, depending on experimental conditions, most CPPs can enter cells through multiple pathways. Although currently there are a variety of experimental methods to explore the proposed CPP internalization mechanism and manifold theoretical models, it is still necessary to verify these findings and investigate the mechanistic details from the perspective of simulation. MD simulations provide insights into these peptide–membrane interactions, help us to analyze the dynamics of internalization processes in depth, provide information on charge, hydrogen bonding, orientation, and helical properties of peptides which can be accurate to specific residues and demonstrate the importance of certain specific residues or events during the penetration process.

### 4.1. Role of Arginine Residues

In general, CPPs have no sequence homologies, and different residues and sequence arrangements may play a role in the process of penetration. This structural diversity leads to different uptake patterns. In most CPPs uptake mechanisms, the usual initial step is the electrostatic interaction of positively charged CPPs with negatively charged membrane components such as phosphate groups, which affects the organization of lipid molecules and leads to membrane perturbation. It has been reported that the effective penetration of CPPs requires at least eight positive charges [58]. It is well known that positively charged arginine residues play an important role in the uptake process, due to the strong interaction between the guanidinium group of Arg and the phosphate groups of lipids, that is, the formation of bidentate hydrogen bonds and electrostatic interactions between the guanidine and the sulfate, the phosphate or the carboxylate moieties of membrane, polyarginine CPPs can cross membrane more efficiently. This strong interaction will destabilize the packing of the lipid and distort the membrane structure, and the charged amino acid residues route water molecules into the hydrophobic core of the lipid bilayer, thereby inducing a hydrophilic pore in the membrane, and facilitating the transfer of CPPs. As we know, antimicrobial peptides are able to translocate across the membrane and form stable pores at the millisecond level during the translocation [24,95]. The arginine-rich CPPs can also form a short-living hydrophilic channel in the plasma membrane of cells upon translocation [96,97,98]. In Ref. [52], Herce and Garcia successfully observed the pore formation caused by polyarginine peptides, and proposed a mechanism for the spontaneous translocation of the arginine-rich HIV-1 Tat peptides across a DOPC lipid membrane. MD simulations in the work showed that on the timescale of 100–200 ns, strong interactions between the Tat peptides and the phosphate groups of the lipid bilayer induced the insertion of charged side chains and the formation of a transient pore, followed by the translocation of the Tat peptides. But the results are controversial because the pore formation in the simulations was observed at a high ratio of peptide to lipid of 1:18 and at elevated temperature of 343 K and 363 K, and meanwhile without counter ions. Particularly suspicious is that the time scale on which peptide penetration occurs is too short compared to experimental observations (on the order of minutes). Yesylevskyy et al. studied the interactions of penetratin and the Tat peptides with DPPC and DOPC phospholipid bilayers by MD simulations at a lower ratio of peptide to lipid of 1:64 [73]. In contrast to the previous simulation work, no transmembrane pores were observed, suggesting that the formation of pores can only be induced above a certain concentration threshold. They further demonstrated that the free energy barrier obtained by the umbrella sampling simulations of inserting a single penetratin peptide into a DPPC bilayer was estimated to be ~75 kJ/mol, suggesting that the spontaneous penetration of a single peptide takes at least a few seconds to a few minutes on a timescale, which is consistent with experimental observations. Huang et al. also demonstrated that water pores can form in the membrane from an energetical point of view [92]. They calculated the free energies of translocating a cycle cationic peptide Arg9 into a neutral DOPC lipid bilayer along two different paths by umbrella sampling MD simulations. The results showed that the free-energy barrier along a pore path was 80 kJ/mol lower than that along a pore-free path. Similar conclusion for the same system was also confirmed in a coarse-grained simulation [67]. Another simulation of penetratin with DPPC lipid bilayer also confirmed that when the penetratin penetrated into the bilayer, a rather significant thinning of the bilayer at the contact site was observed, which may be the first stage of pore formation [99]. Yao et al. studied the transmembrane process of arginine-rich CPP Arg9 and tried to understand the quantitative correlation between the hydrophobic barrier of the plasma membrane and the free energy barrier of the CPP transmembrane using MD simulations and the umbrella sampling methods [100]. The energy analysis showed that multiple salt bridges of guanidinium-phosphate accounted for 65% of the overall interaction energy, whereas the interaction energy from the backbone of the peptide could be negligible, so the increased negative charges of the lipid bilayer or more salt bridges would reduce the transmembrane free energy barrier and further facilitate CPP adsorption and transmembrane processes.

### 4.2. Role of the Secondary Structures of CPPs

It is generally believed that the role of positive charges is critical in the uptake mechanism. The importance of positive charges, especially those of arginine residues, in the uptake mechanism has been demonstrated in [40,101], but the high transport efficiency of TP10, which is lysine-rich and arginine-deficient, suggests that this is not always the case. The work by Magzoub et al. showed that penetratin preferentially interacts with negatively charged membrane components, whereas transportan interacts with membranes independently of charge [102]. In addition, the secondary structures of CPPs, especially the structures adopted when interacting with the membrane, are also important for penetration. The α-helical peptides have proved to be better penetrators than the peptides with random coil structures, especially for pore formation models. Taking the arginine-deficient but lysine-rich Pep-1 for example, CD analysis revealed a transition from unstructured to helical conformation upon increasing the concentration, which is conducive to the insertion of Pep-1 into the membrane by forming a transient transmembrane pore structure [103]. Dunkin et al. used MD simulations to study the interaction between the α-helical amphipathic TP10 and a zwitterionic POPC bilayer [104], and assessed the plausibility of the “sinking raft” model, in which the peptide oriented parallel to the membrane surface, resulting in a mass imbalance across the bilayer, and this perturbation caused the peptide to sink deeper into the bilayer. The results showed that TP10 preferentially binds to the membrane in a parallel orientation, which was energetically favorable because the Lys residues are distributed along the sequence on the same side of the peptide, and this orientation kept the Lys residues in a polar environment during insertion. The simulation results also demonstrated the importance of the amphiphilic structure of TP10, which differs from some peptides (such as Tat) with unstructured conformations at the interface, and the analysis further revealed that the formation of salt bridges between the Lys residues of TP10 and the phosphate groups of the lipids is a key factor in determining the orientation of the peptide as well as stabilizing the peptide–membrane interactions, and the salt bridges can be compared with the Arg-phosphate ones of the HIV-1 Tat peptides determined by NMR [105]. Song et al. further evaluated the effects of structure and charge on the translocation ability of TP10 and its analogs [106]. The results showed that disrupting the helical structure or performing Arg substitution could remarkably reduce the cellular uptake ability of TP10, while increasing the positive charge and the amphipathicity was an effective strategy for enhancing the translocation ability of TP10.

### 4.3. Role of Hydrophobic Aromatic Residues

As for penetratin, which consists of seven basic residues (3 Arg and 4 Lys) and three aromatic residues (1 Phe and 2 Trp), the conformational changes are more complex. Polyansky et al. explored the intramolecular and intermolecular interaction characteristics of penetratin at the water–lipid interface and the conformational plasticity of the peptide by simulating the binding of penetratin to the zwitterionic DOPC and anionic DOPS lipid bilayers [107]. The simulation results showed that penetratin exhibited complex conformational behavior, that is, it maintained helical conformation in an aqueous solution and zwitterionic bilayer, while exhibiting high conformational flexibility in negatively charged membranes. It was also revealed that the conformation of penetratin depended on its initial orientation relative to the membrane, especially when it was in anionic DOPS bilayers. The major contribution of the peptide–membrane interaction was the salt bridges and hydrogen bonds formed by the basic Lys and Arg residues with the lipid polar heads. To evaluate the role of specific intramolecular interactions in penetratin, free energy perturbation (FEP) calculations were performed on aromatic–cationic residual–residue contacts of several model pairs. The results showed that the basic residues Arg and Lys could form energetically favorable pairs with aromatic residues Phe and Trp in the aqueous and apolar environments, which facilitated the insertion of the peptide and enhanced the stability of the membrane-bound state [108]. The formation of the above pairs was important because it was found that the energy gain extrapolated from the FEP calculations might be comparable to that observed upon the creation of backbone hydrogen bonds, which agreed well with the NMR experiment results of penetratin in the membrane mimic media-TFE-water mixture [109] and SDS micelles [110]. Pourmousa et al. also demonstrated the importance of the basic and aromatic residues of penetratin on the binding mode of penetratin to a zwitterionic DPPC lipid bilayer by MD simulations [99]. They found that penetratin exhibited a high degree of conformational flexibility during the simulations, it formed α-helical or β-like conformations in independent different simulation runs, which indicated that penetratin might fine-tune its structure in different membrane surroundings. The authors interpreted that all the different conformations could exist in the penetration process, and the peptide might switch between them when they penetrated into the bilayer. The simulation results also showed that for penetratin, the hydrogen bonding and charge–pair interactions between the bilayer and the Arg and Lys residues could help to localize the charged residues in the hydrophobic region of the bilayer, which was critical for the penetratin binding mode. As for the aromatic residues, Trp could form hydrogen bonds with the phosphate groups of lipids, while Phe formed no hydrogen bonds with lipids.

Other simulations have also observed the contact of basic residues with hydrophobic aromatic residues such as tryptophan during the internalization process of amphiphilic CPPs. Jobin et al. used experimental approaches as well as MD simulations to investigate the membrane insertion of the amphipathic peptide RW16 (RRWRRWWRRWWRRWRR) into the zwitterionic DOPC bilayer and the orientation properties of the peptide during the process [111]. The trend of RW16 insertion was determined by the hydrophobic interaction with the fatty acid chains via Trp residues and the electrostatic interaction with the lipid phosphates via Arg residues. Such interactions are important in the cellular uptake mechanisms because they create a small curvature of the membrane, which can induce the invagination phenomena [112,113] and contribute to the tubulation and internal vesicle formation as induced by RW16 on giant unilamellar vesicles (GUVs) [114]. Moreover, data obtained by NMR spectroscopy and MD simulations both revealed that side chain contacts between Arg and Trp residues contributed greatly to the conformational stability and the biomolecule functions [115,116]. Walrant et al. studied the binding, insertion and orientation of RW9 (RRWWRRWRR) and RL9 (RRLLRRLRR) into membranes [117], where RW9 was internalized into cells effectively, whereas RL9 was not internalized but bound to the cell membrane. During the simulation, the orientation of the peptides changed, and many hydrogen bonds and salt bridges formed between Arg side chains and the lipid phosphate groups, providing anchors for the peptides. It is worth noting that RL9 actually inserted deeper than RW9 and the result was consistent with what was observed experimentally in SDS and DPC micelles. The reason for the difference is that the Leu residues are more hydrophobic and may promote better penetration. However, deep insertion may impair more superficial interactions which could trigger a direct translocation [97], that is, the deeper insertion of RL9 may result in peptide trapping in the membrane. The difference in penetration between RW9 and RL9 might be due to their different effects on lipid packing. RW9 can decrease the lipid chain ordering, allowing lipids to adopt different supramolecular organizations to promote the passage of the peptides, where the Trp residues play a decisive role.

### 4.4. Other Factors Affecting the Uptake Mechanism

There are also several other factors that affect the uptake mechanism, such as initial configuration, changes in CPP conformation, concentration, and length of CPP sequences. On the effect of peptide length, He et al. performed coarse-grained MD simulations to investigate the interaction mechanism between polyarginine peptides with 4, 8, 12, 16 Arg, respectively, and asymmetric membranes including DPPC, DPPE and DPPS [118]. The results indicated that the peptides could penetrate through the lipid bilayer by inducing hydrophilic hole formation. Furthermore, long peptide chain length and high membrane asymmetry level have a positive effect on peptide penetration. In many cases, CPP concentration has shown a significant effect on the pathways and efficiency of penetration. In addition, some studies have shown that polymerized CPPs have better penetration ability than non-polymerized CPPs due to their stronger interactions with the membrane components. Some peptides exhibit their activities depending on the environmental conditions. A typical example is the pH low-insertion peptide (pHLIP), which forms a transmembrane helix and insert into the lipid bilayer under acidic pH conditions, and has shown promising applications in tumor-targeted delivery of cargoes [119,120]. Wei et al. investigated the interaction of a 35-amino acid pHLIP peptide (AEQNPIYWARYADWLFTTPLLLLDLALLVDADEGT) [121] with the POPC bilayers under acidic and basic pH conditions, using all-atom MD simulations with different initial configurations [122]. The results showed that when pHLIP was placed parallel to the bilayer surface, it could insert into the POPC bilayer spontaneously at acidic pH, while at basic pH it bound to the membrane surface without insertion. When pHLIP partially pre-inserted in the POPC bilayer, it could insert deep into the bilayer at acidic pH, while at basic pH it moved toward the bilayer surface.

### 4.5. Comparison and Evaluation of the Characteristics of Different CPPs

In order to compare and evaluate the characteristics of different CPPs during the penetration, Ramaker et al. tested the penetration ability of 474 CPPs from the CPP database for HeLa cells, and the results showed that there were significant differences in penetration efficiency, and none of the CPPs performed best in all conditions [123]. However, CPPs that form stable α-helical structures and have positive charges are good candidates for efficient cargo transport. Kumara et al. investigated the free energy profiles of a single peptide inserting into a neutral DOPC lipid bilayer membrane using coarse-grained MD simulations [124]. The selected 17 CPPs span cationic, hydrophobic and amphipathic categories. The results indicated that the adsorption of hydrophobic peptides was the strongest, and the free energy minima of these peptides were close to the bilayer interface. Among the selected CPPs, K-FGF (AAVALLPAVLLALLAP) acted as the best hydrophobic CPP, consistent with the fluorescence and CD spectroscopy studies [125]. It should be noted that, given the need to release into the cytosol, the strong adsorption of CPPs might be a disadvantage. However, cationic CPPs were found to exhibit only moderate adsorption capacity, that contradicted experimental results in which cationic peptides had been shown to be effective in translocating the membrane, possibly due to the fact that the charge on the bilayer and the concentration of the peptide were not taken into account. The best performers confirmed by experimental results among the cationic peptides were penetratin and R10 (RRRRRRRRRR) [123]. In addition, simulation results showed that Transportan [126] and C6 were the best amphipathic CPPs for passive translocation. In MD simulation studies, the penetration behavior of CPPs is mainly achieved through peptide induced membrane perturbations, resulting in local membrane thinning or the formation of hydrophilic pores. For example, MD simulations of TP10 and its analogs in the DPPC bilayer suggested that the degree of local membrane thinning could reflect the extent of bilayer disturbance, and higher membrane disturbances led to higher translocation activity and higher cytotoxicity [106].

### 4.6. Larger Systems

**Figure 7:**
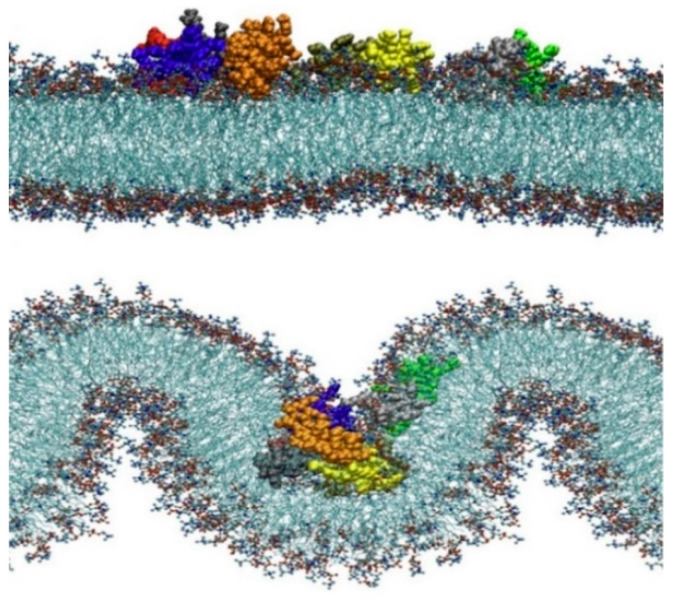
Stages of the deformation of a DPPC bilayer with 512 lipids and 8 bound penetratin peptides. Image reprinted from Ref. [73].

If we adopt larger systems with more lipids, it is hopeful to get richer responses, such as changes in membrane curvature. Yesylevskyy et al. used MD simulations to study the interactions of penetratin and the Tat peptide with the zwitterionic DPPC and DOPC phospholipid bilayers. The extended system consisted of 8 penetratin or the Tat peptide molecules and large bilayer patches including 512 lipids [73]. The simulation results showed that the transmembrane pores did not form spontaneously, but instead the bilayer patches underwent large-scale deformation. The peptides could enter the cell by micropinocytosis, then multiple peptides induced deformations of the lipid bilayer, which subsequently led to the formation of small vesicles that encapsulate the peptides (Figure 7). This mechanism might be mainly attributed to the high charge density on the membrane surface, which was induced by the aggregation of the peptides. Even if this potential micropinocytosis mechanism could be observed, computer simulations of endocytosis are still lacking to date due to the limitations of the temporal and spatial scales of simulations, as well as the fact that endocytosis often requires the participation of membrane proteins. Compared to the pore formation model, the inverted micelle model and carpet model require a larger system, thus at this stage coarse-grained methods might be a better choice for these two models. Kawamoto et.al used coarse-grained simulations to investigate the dynamics of Tat and the bilayer membrane with 512 lipid molecules [127]. They found that the membrane curvatures or invaginations could lead to the formation of inverted micelle when the peptide and the lipid heads were strongly attracted, and the simulation results were in good agreement with the NMR and TEM studies [128]. They therefore proposed a mechanism in which the transition from bilayer to inverted micelle stems from the competition between the attractive potential energy of the peptide and lipid heads and the bending energy of the bilayer.

### 4.7. Design Strategies of CPPs

Although the mechanisms of cellular uptake of CPPs depend on a large number of factors, and the sequences of different CPPs vary greatly (Table 1), there are some similar structural properties that can be referred to in the design of CPPs [129]. Regardless of the type of cargo and the composition of the membrane, the first factor that needs to be considered is the net charge of the peptide. Many CPPs are rich in positively charged arginine residues, and their guanidine groups can form electrostatic interactions, bidentate hydrogen-bonding interactions and salt bridges with the phosphate groups of lipids. Compared with lysine, the guanidine groups on arginine help CPPs cross the membrane more effectively [130]. The second factor is the secondary structure of the peptide [1], especially the structure adopted after the peptide interacts with the cell membrane. For amphiphilic CPPs, the translocation is the result of amphiphilicity rather than positive charge, although most of these CPPs are positively charged. For example, if the MAPs are amphiphically conserved, the cellular penetration ability of neutral MAP17 (QLALQLALQALQAALQLA) and anionic MAP12 (LKTLTETLKELTKTLTEL) can be obtained by replacing lysine with other polar residues [45,131,132], and the results suggest that α-helical structure is more favorable for penetration. The third factor is the peptide concentration, which can trigger different uptake pathways. It is generally believed that amphiphilic peptides penetrate the membrane directly at low concentrations, while non-amphiphilic CPPs are taken up by endocytosis [94]. Besides the above factors, there are other factors that need to be considered when designing CPPs, such as hydrophobicity and the length of the peptide. In addition to the cell penetration properties, we also need to consider the penetration efficiency, the aggregation, and, especially, the cytotoxicity. Therefore, the design of CPPs is a complex task. In contrast, chemical modification of the structure of a known CPP or fusion of effective sequences may be a more economical way. MD simulations of CPP decoration will be discussed in the next section.

## 5. Simulations of CPP Decoration and Coupling

Over the past 30 years, the family of CPPs has expanded rapidly, and the search for new sequences continues. In recent years, numerous studies have reported the potential of CPPs as carriers in drug delivery, and the research field has shifted to the design of peptides and CPP-cargo complexes [129] with higher transduction efficiency, biostability, specificity, and lower cytotoxicity. A comparison between different experiments is relatively difficult due to many factors, such as cell type, cargo type and size, coupling modes of CPP and cargo, and experimental conditions. MD simulations offer a promising approach to facilitate the design of CPPs by decorating or substituting amino acid sequences, fusing valid sequences, or conjugating with small bioactive molecules. For example, improved analogues have been obtained by shuffling the amino acid sequence of penetratin [134]. Although these modified or decorated CPPs have not been accepted for clinical applications, their potential application value has received considerable attention and discussion [135,136].

### 5.1. Chemical Modifications of CPPs

Functionalization or chemical modification of CPPs, such as to include unnatural and modified residues [137] or to substitute the amino acid sequence of CPPs, has been proposed to improve the delivery of cargoes that successfully target specific cells or tissues. MD simulations also reported some studies on the modification or substitution of certain amino acids in CPPs. SAP(E) (VELPPPVELPPPVELPPP) [133] is known as the first synthetic anionic CPP by replacing the positive arginines with glutamic acids from a well-known CPP SAP (VRLPPPVRLPPPVRLPPP). SAP(E) showed similar uptake efficiency and action mechanism to SAP, suggesting that a positive net charge is not a necessary factor for the activity of CPPs. Based on the above reason, Antunes et al. presented a synthetic anionic peptide LE10 (LELELELELELELELELELE), which strongly interacts with model membranes and has demonstrated the properties of CPPs and AMPs both in MD simulations with the POPC and cholesterol bilayers and in in vitro studies with liposomes and mammalian cells [138]. The MD simulations showed that LE10 rapidly interacted with the bilayer surface and led to membrane bending, and at the end of simulations, the membrane involved the peptide, such as one half of a micelle. Simulations of larger systems with a lateral size of 26 nm showed that the curved region of the membrane eventually coalesced into a vesicular bud or a micelle, similar to the early stage of the endocytosis mechanism. Tsai et al. designed the SAP10 (RRWKFFPWRR) peptide, an almost mirror-symmetric peptide derived from the cationic antimicrobial peptide indolicidin (IL) (ILPWKWPWWPWRR), to improve gene delivery safety and efficiency [139]. All-atom MD simulations were performed to understand the association between SAP10 and POPC lipid bilayers. The results showed that the inserted IL and SAP10 peptides exhibited different configurations although they both disturbed the lipid bilayers. Because the cytotoxicity of IL had been reported to associate with Trp residues, the reduction of Trp in the SAP10 peptide resulted in reduced membrane perturbation and improved biocompatibility. In vitro experiments have demonstrated that the designed SAP10 is a safe and effective peptide that promotes PEI-mediated gene delivery. Another example comes from Gimenez-Dejoz’s team [83]. It was reported that the incorporation of nonproteinogenic amino acids, such as α-aminoisobutyric acid (Aib), could increase the helicity, biostability and penetration efficiency of CPPs [140]. They used MD simulations with enhanced sampling technique to study two Aib-containing CPPs, $\mathrm{poly}{\left(\mathrm{LysAibAla}\right)}_{3}$ (KaibA) and $\mathrm{poly}{\left(\mathrm{LysAibGly}\right)}_{3}$ (KaibG), and they both exhibited improved biostability and internalization abilities [141]. Compared to some widely known CPPs such as BP100 and R9, kAibA and kAibG showed the lowest internalization energies due to their compact amphipathic structures, and the Aib residues facilitated internalization by forming hydrophobic contacts with the lipid tails. There are a variety of modification methods, but it is important to note that each modification must be carefully designed to avoid possible problems, such as low solubility, aggregation or toxicity [129,142,143,144].

Another promising approach to improve the efficiency of CPP internalization is to fuse effective sequences, such as the design of the well-known TP10. Selective targeting of CPPS can be achieved through chimerism [66,145,146]. Snini et al. investigated the conformational preference of the fusion peptides Vim-TBS (58-81)-p10 (GGAYVTRSSAVRLRSSVPGVRLLQ-RQTSMTDFYHSKRRLIFS) and Tat (48-60)-p10 (GRKKRRQRRRPPQ-RQTSMTDFYHSKRRLIFS) [147]. These fusion peptides are derived from the Vimetin Tublin binding site (TBS) peptide, the Tat peptide, and a pro-apoptogenic peptide of the p21/WAFI protein, respectively, and the peptide p10 has frequently been used as a model cargo to evaluate cell-penetrating properties. It was found that the above fusion peptides will form helical structures during MD simulations. Compared to the Tat-p10 peptide, Vim-TBS-p10 was predicted to show better cell penetration properties because of its relatively better amphipathic nature and the hydrophilic face formed by the positively charged residues, that facilitated a better interaction with the membrane. The above finding has been well supported by the experimental observations showing that this fusion peptide accumulates and distributes in glioblastoma cells [148]. In the aspect of supramolecular assembly of CPP nanostructures, Ashwanikumar et al. created cell-penetrating self-assembling peptide nanomaterials (CSPNs) with the capability to translocate inside cells through sequential ligation of peptide building blocks [149]. They devised a Tat peptide-based triblock array, in which the Tat peptides were conjugated to an amphiphilic linker ${\left(\mathrm{RADA}\right)}_{2}$, followed by the sequential addition of Phe residues. This design enhances the hydrophobicity of the CSPN, and makes it a versatile host that can accommodate guest molecules. The designed CSPNs containing two, three or four Phe residues would self-assemble into “nanodrill-like structures”, which had high capacity to encapsulate hydrophobic guest molecules and enhanced the internalization. MD simulations of the $\mathrm{Fmoc}-\mathrm{FF}-{\left(\mathrm{RADA}\right)}_{2}-\mathrm{Tat}$ (2F-RT) peptide aggregate were also carried out in the work to study the evolution of secondary structure and intermolecular interactions. During the simulation, the Tat segment lost its α-helical nature and formed random coils, while the amphiphilic ${\left(\mathrm{RADA}\right)}_{2}$ retained its native helical structure for the most part. In addition, the peptides in the core of the aggregate fluctuated significantly less than their surface counterparts, which allowed them to form intermolecular salt bridges. From this work, we can see that MD simulations provide microscopic insights into the structural properties and mechanisms of action of fusion peptides, which is of great significance for guiding and facilitating the design of peptide drugs with cell penetration functions.

### 5.2. CPP Coupling with Cargoes

Designing a perfect analytical method to characterize the transport dynamics of CPPs and cargoes is a difficult task because the internalization process of CPPs is very complex and strongly depends on the type, size and binding method of the cargo molecules. In different experiments, CPP might function as a monomer or form complex with its payload molecule. For the latter, CPPs can form complexes with cargo molecules through covalent or non-covalent bonds. In the case of covalent conjugation, CPPs are coupled to neutral cargoes via disulfide bonds, amide bonds, etc., while in the case of non-covalent conjugation, CPPs are coupled to negatively charged cargoes by electrostatic and/or hydrophobic interactions. The conjugation of peptides offers a possible pathway for the design of new drug delivery systems and thus holds great potential in future treatments. For the CPP-dominated cargo delivery system, the internalization efficiency can be changed by modifying the properties of the cargoes and their binding methods. Alfonso T. Garcia-Sosa et al. studied the stability and binding affinity of the complexes of CPPs NF51, PF3, PF6, and TP10 to siRNA [150]. In the work, they found that the bound structures of all complexes were stable throughout the MD simulations, and the stability and binding affinity were related to the sidechains and modifications of the CPPs. The guanidino groups on arginine residues might bind to nucleic acids through electrostatic, hydrogen bonding, cation-π, and π-π interactions. The stearyl groups presented in PF3, PF6, and NF51 and the quinoline groups presented in PF6 increased the binding affinity and stability of their complexes with siRNA compared to TP10, which lacks these groups. CPPs with conjugated fatty acid moieties have shown excellent potential as novel delivery excipients as the increased hydrophobicity of the CPP carriers promotes stronger association with the lipophilic cell membrane [151]. Furthermore, this modification of CPPs might increase the self-assembling tendency of the peptides and then diminish the interactions with the membrane. To explore the impact of site and degree of lipidation on the membrane interactions of a cationic CPP, Hedegaard et al. designed four peptides lipidated on different lysine residues using capric acid (C10) based on the penetratin analogue PenetraMax (KWFKIQMQIRRWKNKR) [152]. Experimental results and coarse-grained MD simulations demonstrated that all CPP conjugates interacted with the membrane by inserting lipid chains into the core of the bilayer, and that the site and degree of lipidation affected the behavior of the carriers in their interactions with the membrane and thus altered the penetration efficiency of CPPs. In addition, membrane thinning effects and CPP induced membrane bending were also observed. The work revealed that the CPP performance could indeed be tuned by lipidation to facilitate membrane insertion. In most experimental studies, CPPs are tethered to fluorescent dyes in order to allow tracking of peptide transduction events under the microscope. Studies have shown that different fluorophore labeled peptides may also affect uptake mechanisms, intracellular distribution and cytotoxicity. To explore the possible role of dyes in assisting translocation, Akhunzada et al. used MD simulations to analyze the Tat peptides in monomeric and dimeric forms across a purposely-created membrane pore, whether or not covalently-linked to the TARMA dye [153]. The results showed that in comparison to the monomers, Tat dimers translocated much more slowly, thus their capability to stabilize membrane pores was enhanced due to the strong coulomb interactions between arginine residues and surrounding lipid head groups. Combined with other studies, a picture for the role that the TAMRA dye played in the process of Tat11 membrane transduction was drawn: it promotes peptide dimerization and, by doing this, it remarkably slows down the translocation kinetics, thus enhancing the lifetime of the membrane pores.

**Figure 8:**
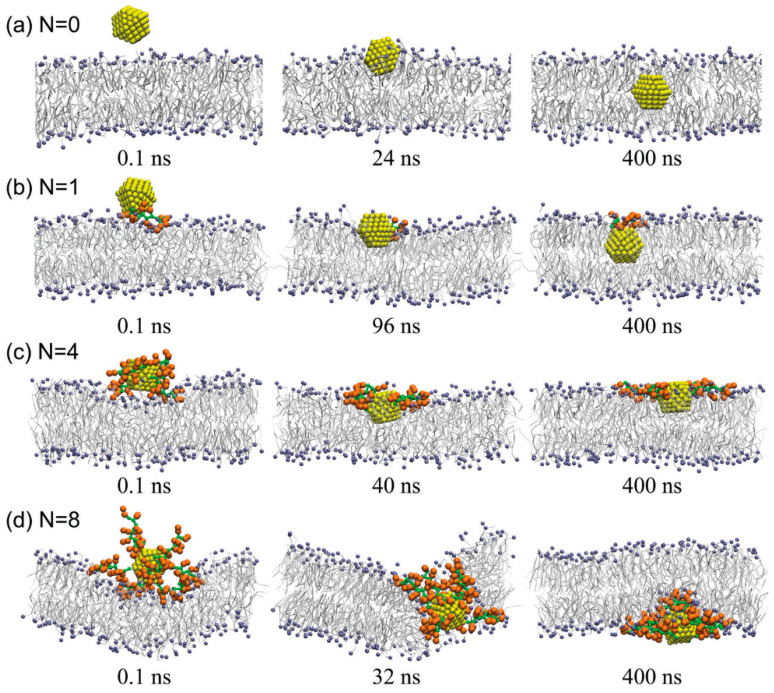
Representative snapshots of (**a**) the unmodified AuNP (N = 0) and (**b**–**d**) the Tat–AuNP complexes (N = 1, 4 and 8) interacting with the lipid membrane (N denotes the peptide number). Image reprinted from Ref. [157].

The coupling of CPPs with nanoparticles has also been studied by many MD simulations. Using coarse-grained MD simulations, Hu et al. showed that the transmembrane efficiency of polyarginine (R8) peptides was improved by conjugating with small hydrophilic nanoparticles (NPs) with appropriate linkers [154]. At high concentrations, the R8-NP conjugates could directly translocate across the asymmetry membrane through water pores. The translocation efficiency was closely related to the length of the linkers. When the linker length was about half of the membrane thickness, the translocation efficiency reached a maximum. However, the R8-NP conjugates with overlong linkers not only reducing the transmembrane efficiency due to the blockage of NPs in the water pores, but also causing cytotoxicity because the water pores were not closed for too long. Magnetic iron oxide nanoparticles have also been actively investigated for their possible applications in medicine, as they represent the next generation of targeted drug delivery technologies, and silica is the most common compound for the preparation of coated iron oxide nanoparticles. Grasso et al. investigated the adsorption of penetratin on silica and magnetite (MAG) surfaces, and estimated the penetratin-surface adsorption free energy quantitatively by umbrella sampling MD simulations [155]. The results indicated that Arg and Lys played a key role in surface binding, despite their physicochemical properties and surface charge densities differ, and this result was consistent with similar computational studies focusing on a spontaneous membrane penetrating peptide (PLIYLRLLRGQFC) adsorption to the silica surface [156]. The simulation study from Grasso’s team suggested a competitive mechanism between magnetic nanoparticles (MNPs) and cell membranes, both of which interact with the same sites of penetratin and may partially inhibit penetratin from carrying out its membrane penetrating function. In order to overcome the difficulty of low cellular internalization efficiency of gold nanoparticles (AuNPs), decorating AuNPs with functional moieties such as CPPs has been considered as a promising strategy. Quan et al. explored the interactions of the Tat peptides and their conjugated AuNPs with lipid membranes by coarse-grained MD simulations [157]. The results showed that the translocation of AuNPs across the membrane was significantly affected by the Tat concentration on the particle surface (Figure 8). When a small amount of peptides were decorated on the AuNP surface, the transmembrane efficiency of AuNPs might even be reduced, because the peptides adhering to the outer membrane surface might restrict the movement of AuNPs into the membrane. However, when the number of grafted peptides increased to a threshold, a transient hydrophilic pore formed in the lipid membrane due to the transmembrane electrostatic potential difference, so that the Tat-AuNP complex could translocate across the membrane in a pore-mediated way.

### 5.3. The Design of Self-Complementary Peptides

It is also noted that ionic-complementary peptides can be modified to exhibit similar properties to CPPs by adjusting the amino acid sequence and chain length. As a new type of nanomaterial with wide application value, the side chains of ionic self-complementary peptides are complementary to each other, that is, positively charged and negatively charged amino acid residues can form ionic pairs. This special structure determines that they can spontaneously assemble to form stable nanostructures [158], and the self-assembly is mainly driven by intra- and intermolecular hydrogen bonding, hydrophobic, and electrostatic interactions. In previous work, we performed MD simulations to investigate the adsorption and self-assembly behaviors of the amphiphilic ionic complementary peptide EAK16-II (AEAEAKAKAEAEAKAK) on the hydrophobic highly ordered pyrolytic graphite (HOPG) surface [159,160]. Simulations showed that the peptides tended to form hydrophobic contacts with the HOPG surface rather than to form a hydrophobic core, and the solution pH could affect the adsorption rate of EAK16-II. Under neutral pH condition, the interchain electrostatic attraction was favorable for adsorption, while under acidic and basic conditions, the electrostatic repulsion force slowed down the adsorption because of the protonation and deprotonation of glutamic acid and lysine residues. Hydrogen bonds did not play an obvious role during the adsorption, but several long-lasting interchain hydrogen bonds were observed after the peptides depositing on the surface, indicating that hydrogen bonds played an important role in the subsequent peptide self-assembly process.

**Figure 9:**
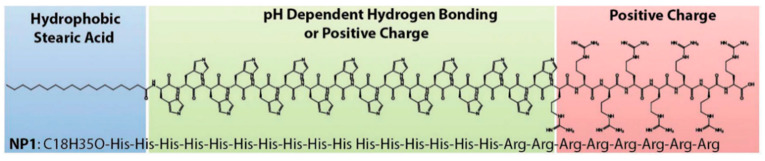
Molecular structure of NP1 which contains a hydrophobic stearic acid, histidines and positively charged arginines. Image reprinted from Ref. [163].

Molecular self-assembly is a widespread phenomenon in nature. Self-assembling peptides are a kind of biomaterials that are relatively easy to design and synthesize, and have many potential applications such as templates for nanowire fabrication and delivery of peptide medicines. Studies have shown that abnormal folding and aggregation of proteins can lead to related diseases, such as Alzheimer’s, Parkinson’s, Down’s syndrome, and the nanostructure formed by peptides depends on the influence of the amino acid sequence and solution pH [161]. By regulating and modifying the amino acid sequences of peptides, the nanostructures generated by self-assembly of peptides can be controlled, which can be used in the design and application of new nanomaterials. Chen et al. proposed a de novo amino acid pairing (AAP) design principle, that is, using the hydrogen bond pairing, ionic and hydrophobic pairing strategies to construct new self-assembled peptides (SAPs), which could self-assemble into β-sheet rich nanofibers [162]. This kind of peptide showed the ability to stabilize and deliver hydrophobic anticancer agent ellipticine in an aqueous solution. Moreover, the peptide–drug complexes exhibited good antitumor activity against human lung carcinoma cells A549 and breast cancer cells MCF-7. For example, they put forward a systematic design of a histidine-rich lipidated peptide sequence named NP1 (Figure 9), which contains a hydrophobic stearic acid, histidines and positively charged arginines, where histidines promoted the proton sponge effect and arginines had a high affinity to bind to the cell surface for intracellular delivery [163]. The NP1 peptide successfully exfoliated graphite flakes, exhibited long-term dispersion stability in aqueous solution, and functionalized the graphene nanosheets, which not only exhibited good biocompatibility with cells, but also enhanced cell uptake of cancer drugs. The details of peptide–peptide and peptide–graphite interactions were investigated by experiment and all-atom MD simulations. MD results showed that the strength of the repulsive interactions might cause a single-layer peptide pattern with ∼0.2 nm height, and a multi-layered (or compact) structure with ∼3.1 nm height under the acidic and neutral conditions, respectively, as observed in AFM images. In another study, they tried to modulate the morphologies and internal cohesion of peptide amphiphile (PA) self-assemblies and their resultant functions by tuning the molecular interactions [164]. The coarse-grained MD simulations were applied in the work and revealed that Palm-CR3 (C16-CSNSNSRRR) self-assembled into cylindrical supramolecular fibers in water with a diameter around 8.0 nm, whereas the fiber diameter decreased to around 7.0 nm in a high ionic strength environment. Around the positively charged surface of the fiber, abundant chloride ions were observed while sodium ions were rare, resulting in the screened electrostatic repulsion. When electrostatic repulsion was screened, the peptide amphiphiles were supposed to interact with one another in a more compact manner than their “repulsion on” counterparts. The widths of Palm-CR3 fibers measured from the TEM images confirmed the decrease of fiber diameter when electrostatic repulsion is switched off. The simulations also found that the n-dodecyl-β-D-maltoside (DDM) surfactant molecules interacted with Palm-CR3 molecules and took part in the formation of variform clusters.

## 6. Simulations on Membrane Modification and Simulations of Multi-Component Membranes

### 6.1. The Effect of the Membrane Compositions on CPP Uptake Process

Besides the physicochemical properties and concentrations of the CPPs themselves, other factors that affect the uptake pathways of CPPs are the composition and properties of the membrane. The cell membranes constitute the first barrier for molecules and ions to enter the cells. Lipid membranes have complex and heterogeneous structures composed of diverse components such as different lipids, head groups, membrane proteins, and play an important role in the normal function of cells. Normally, the lipid bilayer can be divided into the choline group (head), the phosphate group (PO4), the glycerol ester group (GE), and the hydrocarbon chain (HC). Such a structure leads to the complexity of interactions between the membrane and CPPs and also affects the way CPPs are inserted. For example, negatively charged components of the membrane, such as phosphate groups and glycosaminoglycans, contribute to the accumulation of positively charged CPPs on the membrane surface, leading to the membrane destabilization, allowing the peptides to traverse the membrane. Mukherjee et al. studied eight different model membranes containing zwitterionic POPC, anionic POPG and POPS, and neutral POPE, as well as various heterogeneous lipid bilayers, to understand the effect of lipid composition on the antimicrobial peptide MSI-594 (GIGKFLKKAKKGIGAVLKVLTTG) using accelerated MD simulation, and confirmed that the lipid composition indeed affected the peptide–membrane interaction [165]. During internalization, the formation of pores is associated with a non-trivial free energy cost, Hu et al. used umbrella-sampling MD simulations with a lipid-density-based order parameter to investigate the membrane-pore-formation free energies of cyclic nona-arginine peptide Arg9 in 18 different lipid bilayer systems [166]. The lipid bilayer systems in the work are classified into three broad classes based on the length, the saturation of hydrocarbon acyl chain and the charge state of the lipid headgroup. The results showed that nona-arginine could induce transmembrane pores, and the free energy of peptide translocation along the pore pathways was relatively lower than that of the pore-free pathways. It was also revealed in the work that the pore-formation barriers were dependent on area per lipid, lipid bilayer thickness, and membrane bending rigidities. $\mathrm{Niemel}\ddot{a}$ et al. also studied the properties of three-component lipid bilayers, so-called lipid rafts, rich in cholesterol (CHOL) and sphingolipids using MD simulations [167]. Their work showed that the membrane elasticity and dynamic properties were strongly dependent on lipid composition due to the local interactions between lipid species. The lateral pressure profile of the membrane could be altered by changes in lipid content, which in turn regulated the function of some membrane proteins. Another work by Crosio et al. showed that the peptide accumulation on the membrane surface was maximal for the DOPC/CHOL/DOPG composition [61]. However, the peptides did not insert into the monolayer containing CHOL, indicating that although the anionic membrane concentrated CPPs on its surface, the reduced permeability prevented CPP translocation. The PMF calculated from umbrella sampling simulations using the coarse-grained force field revealed that the translocation free energy barriers decreased synchronously as DPPS composition increased due to the favorable peptide-anionic lipid interactions. Moreover, the addition of CHOL into the membrane increased the barriers of peptide translocation. CHOL not only coordinated with the peptide leading to a decrease in the peptide-lipid interactions, but also stabilized the liquid-ordered phase of membranes, which would suppress the reorientation of the lipid molecules and lead to the increase of the elastic stiffness of bilayers. Consequently, cholesterol hindered the formation of transmembrane pores, which was in consistent with experimental observations [168]. Similar conclusions were also obtained in MD simulations of the HIV-1 Tat peptide translocation in DPPC/DPPS membranes with the presence of 0–30 mol% cholesterol [169], that is, the cholesterol stabilized the liquid-ordered phase of the membrane and increased the elastic stiffness of the bilayer, thereby hindered the formation of transmembrane pores. Recent work by Tang et al. provides another perspective on the role of cholesterol [170]. The plasma membrane of cancer cells is rich in cholesterol, which impairs T-cell mediated cytotoxicity because cancer cells soften cortical structures through cholesterol enrichment in the plasma membrane. Cancer cells can be stiffened through cholesterol depletion, thus enhancing the cytotoxicity of T-cell and the efficacy of adoptive T-cell transfer (ACT) therapies. The results show the importance of the composition and mechanical properties of the plasma membrane, which are worthy of further study from the perspective of simulation. Polyansky et al. used MD simulations to investigate the spatial heterogeneous distribution of hydrophobic/hydrophilic “mosaic” character of the bilayer surface and their influence on the binding mode of penetratin [171]. They calculated and mapped the molecular hydrophobicity potential (MHP) which has been used to characterize the polarity of peptide–membrane interactions and found that the polar properties of the local environment of peptide residues at the interface could vary significantly, determining the detailed characteristics of the insertion. In anionic membranes, the initial adsorption of penetratin was strongly depended on the “complementarity” between the polar properties of the peptide and its local interfacial environment. If a high degree of complementarity was established, penetratin would penetrate deeply into the membrane without significantly disrupting its initial secondary structure. Such effects explained the complicated behaviors of CPPs, especially if the target membrane surface was of distinctly mosaic nature, and depending on the microscopic properties of the water–lipid interface, the peptides were able to adopt different pathways to exert their biological activities.

Since lipids in the outer membrane of eukaryotic cells are mainly zwitterionic, and the membranes have anion properties owing to the presence of glycosaminoglycans, for the choice of membrane models, most MD simulation studies on the interactions of CPPs with membranes employed simplified anionic or zwitterionic lipid bilayers in the past. For example, Crosio et al. investigated the effect of mechanical properties of anionic membranes with different fluidity and rigidity on the adsorption and penetration of poly-arginine [61]. With the improvement of computing methods and the development of computing resources, computational models of cell membranes have undergone a transition from simple models to multi-component models, such as mixed bilayers with different compositions [172,173]. Efforts are being made to realize more and more realistic models in which different groups in lipids can be distinguished [69], as well as models that can be used for larger systems. For example, Wang et.al proposed an implicit solvent coarse-grained model for quantitative simulations of POPC bilayers [174], by matching the structural and mechanical properties from experiments and all-atom bilayer simulations, the interactions were systematically adjusted to make the model more useful for studies of large-scale phenomena in membranes.

### 6.2. The Effect of Membrane Tension and Transmembrane Potential

Additional constraints or forces can also be applied to membranes to observe the effect on CPP penetration. For example, in many cases, osmotic pressure and external forces can stretch the membrane and position it under tension. He et al. used coarse-grained MD simulations to study the translocation of the polyarginine peptides Arg8 across the asymmetric human erythrocyte membranes, including DPPC, DPPE and DPPS lipids under tension [175]. The results showed that the membrane tension could inhibit the penetration of peptides, and with the increase of membrane tension, the inhibition effect strengthened as the membrane became thinner, the lipid tails were more disordered, and the potential of intertwining between neighboring lipid tails increased. Higher concentrations of CPPs or the application of a transmembrane potential could destabilize the membrane, thereby promoting the internalization of CPPs. Gao et al.’s MD simulation work provided evidence for the direct translocation of CPPs across membranes driven by membrane electrostatic potentials, which are ubiquitous in biological systems [176]. The local membrane potential could be generated by an imbalance in the concentration of transmembrane ions, and if positively charged CPPs adsorbed on the membrane, the membrane potential would further increase, resulting in the opening of membrane pores through which CPPs could be transported. The authors applied the classical nucleation theory to estimate the translocation time by calculating the changes in free energy when CPPs transferred across the membrane at different potentials, and the results were in good agreement with experimental measurements [177].

### 6.3. Peptide-Induced Membrane Response

Another issue of concern is the possible membrane response to CPP binding, including peptide-induced changes in membrane structure, such as membrane thickness, curvature, area per lipid, the lipid rearrangements, lateral diffusion constants of lipid molecules, and membrane mechanical performance. These parameters are important for understanding the peptide-lipid interactions. For example, the order parameter represents the mobility of the C-H bond of the lipid tails, and is calculated by averaging over time and all lipid tails. During interaction with CPPs, peptide-induced membrane responses could reflect some important aspects of the CPP penetration mechanism, such as the insertion of polar side chains into the hydrophobic core of the membrane to induce local membrane deformations [178,179], which had been thought to be a possible translocation mechanism for Arg-rich peptides. To understand how CPPs affect the mechanical properties of membranes, Grasso et al. used MD simulations to investigate the interactions between different types of CPPs embedded in the DOPC lipid bilayers [180]. The findings highlighted that the presence of CPPs increased local lipid disorder caused by the amount of water molecules conveyed by the peptides within the lipid bilayers, and the lipid disorder was directly related to the membrane stiffness. As a consequence, water penetration promoted by CPPs led to a local decrease in lipid order, which emerged macroscopically as a decrease in the modulus of membrane bending. Meanwhile, other simulations also found that the phenomenon of membrane bending is concentration dependent [138]. However, there’s still a lot that’s unclear for us to understand the membrane response to CPP binding, more research efforts need to be made in this field.

## 7. Conclusions and Future Perspectives

Cell-penetrating peptides have become promising candidates for next-generation intracellular drug delivery carriers due to their ability to facilitate the transmembrane transport of cargo molecules. However, how to improve the transmembrane efficiency and selective permeation of CPPs while limiting their cytotoxicity is a long-standing challenge for scientists. Considering the complicated physiological environment, the factors affecting the penetration process and internalization efficiency are not isolated. On one hand, the transmembrane efficiency depends on the choice of membrane model; on the other hand, the insertion of CPPs leads to changes in membrane thickness, curvature, etc., or the formation of hydrophilic pores, which in turn affects the internalization process. In addition, the effect of cargo molecules on the internalization is critical, and their physicochemical properties may greatly affect the performance of CPPs. In this review, the summarized computational methods have been shown to provide feasible pathways for elucidating the details of peptide–membrane interactions. All-atom MD simulations provide atomistic information on the intra- and inter-molecular interactions that drive peptides binding to the water-lipid interface, and characterize the cooperative mechanisms of membrane destabilization and the thermodynamics of these processes. Such details are especially complementary to experiments and are helpful in guiding the design of new experiments. We also introduced the research progress of MD simulation methods on CPP-membrane interactions in this review, including MD simulation methods and techniques, CPP penetration mechanisms, decoration or coupling of CPPs, the effect of different membrane models on the penetration process, and peptide-induced membrane reactions, as well as the comparison of simulation and experimental results. Investigating peptide–membrane interactions through MD simulations can let us focus on the influence of one or more specific factors, help us to understand the translocation mechanism at atomic scale, and provide new insights into designing more efficient translocation sequences.

For the mechanisms of CPP internalization, most of the simulation results can be explained by the pore formation model. Many CPPs are rich in positively charged arginine residues, and their guanidine groups can form electrostatic interactions, bidentate hydrogen-bonding interactions, and salt bridges with the phosphate groups of lipids. Such strong interactions destabilize the packing of the lipids and distort the membrane structure, and the charged amino acid residues will route water molecules into the hydrophobic core of the lipid bilayer, thereby inducing a hydrophilic pore in the membrane, and facilitating the transfer of CPPs. For some amphiphilic CPPs lacking arginine, peptides with the α-helical conformation have been shown to have better penetration when interacting with the cell membranes, especially in the pore formation model. In some simulations, the peptides are oriented parallel to the membrane surface, resulting in a mass imbalance across the bilayer, and this perturbation causes the peptides to sink deeper into the bilayer, which can be explained by the membrane thinning model. If we adopt larger systems with more lipids, it is hopefully to obtain richer responses, such as changes in membrane curvature. When the peptides and the lipid heads are strongly attracted, the membrane curvatures or invaginations may lead to the formation of inverted micelles. Some related simulations have shown that the aggregation of peptides induces the deformation of the lipid bilayer to form small vesicles that encapsulate the peptides, and the bilayer patches undergo large-scale deformation, which might be a potential microendocytosis mechanism. Computer simulations of endocytosis are still lacking to date due to the limitations of the temporal and spatial scales of simulations, as well as the fact that the endocytosis often requires the participation of membrane proteins.

More efforts are being made to improve the application availability of CPP simulations. Studies on the translocation mechanism of CPPs have shown that the secondary structures of CPPs and specific residues, such as positively charged arginine and lysine, as well as aromatic amino acids are of significant importance during uptake. Identifying intrinsic properties and conformational preferences of the peptides by MD simulations provides valuable clues for more flexible engineering design of novel CPPs. Minor modifications to the CPP structure may significantly alter its cellular uptake capacity, as this bottom-up approach enables regulation of the secondary structure of the peptide, making it promising for clinical applications such as cancer imaging and therapy. The optimization of the structure can be achieved by sequence recombination or amino acids substitution of the widely studied CPP templates. The affinity and stability of ligand docking and the transport efficiency of the complexes can also be improved by modifying the nature of the cargo and the CPP-cargo coupling mode, which is another effective way to design novel peptide-based drug delivery systems with high translocation efficiency and low biotoxicity. Studies have shown that the presence of cargoes affects the internalization pathway of CPPs, and the coupling with cargoes also alters the chemical characteristics of the CPPs, which in turn alters their interactions with the membrane.

In the end, some limitations should be addressed for the applications of MD simulations. Due to the limited accuracy of force fields, the difficulty in adequate sampling of the full energy landscape, the simplification of the membrane model, and the convergence problem, the interpretation of simulation results must be very cautious. The outcome of the simulations depends largely on the initial conformations and orientations of the peptides, the extent of equilibration, and the conditions under which the simulations are performed. It is also possible to miss the lowest free energy translocation path due to inappropriate CV selection, resulting in sampling of a local minimum energy region. Therefore, it is necessary to perform the simulations starting from the significant conformations obtained in experiments such as NMR, X-ray crystallography and cryo-electron microscopy, and to run multiple MD trajectories under independent start-up conditions to understand the average behavior, and the convergence of the simulations must be evaluated carefully. One possible approach is to incorporate experimental data into model development to make the simulation models more quantitative consistent with experiments. With the development of new sampling methods, more accurate force fields and membrane models, it is now promising, with the help of MD simulations, to extract the structure-activity relationship of CPPs, to adjust the nature of existing peptides and the coupling mode with cargo molecules, and to predict and generate bioactive CPPs with high transmembrane efficiency and cell selectivity.

## Figures and Tables

**Table 1:** Overview of some representative CPPs and their properties.

Peptide	Sequence	Type	Number of Arginine Residues	Charge	Conformation *	Concentration Dependence **	Ref.
HIV-1 Tat	GRKKRRQRRRPPQ	Cationic	6	+8	random coil	+	[26]
Arg9	RRRRRRRRR	Cationic	9	+9	random coil	+	[40]
Penetratin	RQIKIWFQNRRMKWKK	Cationic and Amphipathic	3	+7	α-helix or β-strand	+	[30]
TP10	AGYLLGKINLKALAALAKKIL	Amphipathic	0	+4	α-helix	+	[35]
pVEC	LLIILRRRIRKQAHAHSK	Amphipathic	4	+8	β-strand or random coil	+	[41]
Pep-1	KETWWETWWTEWSQPKKKRKV	Amphipathic	0	+3	α-helix	+	[43]
CADY	GLWRALWRLLRSLWRLLWRA	Amphipathic	5	+5	α-helix	+	[38]
MAP	KLALKLALKALKAALKLA	Amphipathic	0	+5	α-helix	+	[39]
SAP(E)	VELPPPVELPPPVELPPP	Amphipathic	0	-3	α-helix	-	[133]
K-FGF	AAVLLPVLLAAP	Hydrophobic	0	0	random coil	+	[44]

* Conformation: conformations adopted when CPPs interact with cell membranes. ** Concentration dependence: whether the uptake mode of CPPs is concentration-dependent, “+” indicates concentration-dependent and “-” indicates concentration-independent.
